# Development and characterisation of charcoal briquettes from water hyacinth (*Eichhornia crassipes*)-molasses blend

**DOI:** 10.1371/journal.pone.0207135

**Published:** 2018-11-09

**Authors:** Naomi P. Carnaje, Romel B. Talagon, Jose P. Peralta, Kalpit Shah, Jorge Paz-Ferreiro

**Affiliations:** 1 School of Technology, University of the Philippines Visayas, Miagao, Iloilo, Philippines; 2 Institute of Fish Processing Technology, University of the Philippines Visayas, Miagao, Iloilo, Philippines; 3 School of Engineering, RMIT University, Melbourne, Victoria, Australia; Assam University, INDIA

## Abstract

Charcoal briquettes are inexpensive solid fuels made from carbonized biomass. The potential of converting water hyacinth (*Eichhornia crassipes*) charcoal into briquettes with molasses as binder was investigated in this study. Dried water hyacinth was carbonized at a temperature between 350°C to 500°C in a fabricated fine biomass carbonizer. A solution containing 80% by weight molasses was used in the production of briquettes having different charcoal/molasses ratios of 40:60, 30:70, and 20:80. Each briquette was characterized in terms of bulk density, calorific value, compressive strength, proximate analysis and micro-structure by Scanning Electron Microscopy. Charcoal briquettes were tested for their flammable characteristics through their burning rates and ignition time. Altering the molasses to charcoal ratio affected the quality and characteristics of the briquettes. Volatile combustible matter and fixed carbon increased with increasing amount of binder while ash content decreased. The 30:70 charcoal/molasses ratio produced the highest calorific value (16.6 MJ/kg) and compressive strength (19.1 kg/cm^2^). The results have shown the potential of converting water hyacinth into an alternative fuel source.

## Introduction

Large volumes of waste biomass residues are generated annually in developing countries as by-products of the commercial forestry, agricultural and industrial sectors. Energy in the form of firewood and charcoal has been the most conventional source of renewable energy in developing countries and has traditionally been used to dispose of these wastes. In the Philippines, about 30–50 million metric tons of wood fuel and biomass residues are estimated to be consumed per year [1]. This extensive use of fuel wood can pose environmental threats to forest trees and contribute to erosive processes. Moreover, fuel wood and biomass residues have low combustion efficiency, also posing environmental and health hazards [2].

On the other hand, the utilization of biomass waste or residue as energy source could help alleviate dependence on imported energy and its use continues to be a topical issue in both developing and developed countries.

In order to upgrade biomass residues for a variety of applications, their original form characterized by high moisture content, irregular shapes and sizes, low bulk density, difficulty in handling, transporting and storing, have to undergo some changes to make their use more practical and economical [3,4]. Some of these drawbacks can be overcome through densification of biomass residues with appropriate binders for briquette production. Several studies report production of briquette from rice husk blended with corn cobs and starch solution binder [5], rice husk and bran with binders cassava wastewater, and okra stem gum [6], bagasse, clay and molasses [7], wood charcoal bonded with arabic gum and cassava starch [8], low rank coal and sawdust [9].

Water hyacinth (*E*. *crassipe*s) is a native plant in the Amazon basin, but it is considered a highly invasive aquatic weed, infecting dam, lakes and irrigation channels in most tropical and subtropical regions. One major problem associated with water hyacinth is its rapid growth rate. It can easily adapt and compete with other aquatic plants causing a major threat to the aquatic environment [10].

Excessive amount of water hyacinth in the aquatic system can reduce biodiversity, displace native species, damage hydroelectric systems, and affect water quality and flow. When not managed and controlled, these plants will cause blockage in bodies of water, resulting to floods during heavy rains and typhoons. Philippine waters that had been damaged by water hyacinth are the Pasig River in Manila and Dansalan River in Datu Piang Maguindanao.

Although water hyacinth is seen by many countries as a weed and is responsible for many environmental and health problems, much research has been done in order to find useful applications for these plant. This includes as soil amendment after composting [11] or for the removal of heavy metals from aquatic systems [12]. Another of these applications is fuel production. Aerobic/anaerobic digesters is a well-established technology that can produce biogas at a relatively low cost and higher yields [13]. Njogu et al. [14] reported biogas production containing about 46 to 53% methane (CH_4_) from water hyacinth -cow dung mixture. Yields of biogas are generally low when not hybrid mixtures of organic matters are used [13].

Conversion of water hyacinth to charcoal dust via pyrolysis has been reported in several studies as potential source for the production of locally needed fuels [15]. To improve fuel quality, the charcoal has to undergo densification or briquetting. This is to increase strength, durability and reduce cost of transportation, handling and storage [3]. Limited studies however, have been conducted to determine the effectiveness of binding methods and to determine combustion characteristics with different binders. A study by Koser et al. [16] showed the technical feasibility and economic viability of densified water hyacinth and cotton stalks. Supatata et al. [17] characterized fuel briquettes from water hyacinth with sewage sludge as binder. Other binders used in previous studies are palm oil mill residue and cassava flour [15], cow dung [18], and starch [19].

Briquette properties are affected by the proportion of binders [9]. However, many studies have used a single ratio (see Table 1). One of the problems commonly encountered in the use of charcoal and briquettes from biomass wastes is their difficulty to ignite [20]. This study aims to characterize the optimum ratio of molasses to charcoal in order to produce fuel briquettes with a high calorific value and rapid ignition time. A range of analytical techniques were used in order to perform a mechanical (bulk density and compressive strength), thermal (calorific value and ignition time) and morphological characterization (FITR and SEM).

**Table 1:** Comparison of FC, VCM, moisture, ash of water hyacinth-molasses briquette with briquettes produced from other sources of biomass and binder.

Briquette type (biomass-binder)	Biomass Binder ratio	Fixed C (%)	VCM (%)	Moisture (%)	Ash (%)	Higher Heating Value (MJ/kg)	Reference
Coal-sawdust (C-S)	50:50	79.5	11.0	1.7	7.83	32.1	[9]
Coal-sawdust (C-S)	33:66	85.0	7.8	0.9	6.3	31.6	[9]
Coal-sawdust (C-S)	25: 75	85.9	7.3	1.1	5.7	32.0	[9]
Neem wood-starch	1:3	85.2	10.5		4.2	32.8	[8]
Neem wood-starch	5: 2	84.25	10.0		5.7	32.3	[8]
Wood sawdust-cow dung	70:30	11.1	75.7		14.9	28.6	[37]
Rice husk(RH)-rice bran bran(RB)–water	10% RB					16.0–16.4	[6]
Bagasse-clay-molasses	40:1:1	36.4	27.2	4.1	36.4	18.4	[7]
WH-phytoplankton scum(PS)	50:50					17.91	[32]
WH-EFB-Cassava starch	25: 75	15.97	80.3	9.3	15.97	17.17	[15]
**WH-molasses**	**40:60**	**13.2±1.3**	**45.8±0.6**	**14.6±0.3**	**26.2±1.0**	**15.9±3.9**	**This study**
**WH-molasses**	**30:70**	**15.0±1.2**	**47.4±1.5**	**18.1±0.1**	**19.5±2.6**	**16.6±2.1**	**This study**
**WH-molasses**	**20:80**	**16.8±0.8**	**48.0±2.2**	**18.6±0.2**	**16.5±2.3**	**13.45±1.6**	**This study**

## Material and methods

### Collection and preparation of water hyacinth sample

Dumangas is a municipality in the Province of Iloilo in Western Visayas, Philippines. Dumangas lies at the tail-end of one of the largest rivers in the Province of Iloilo, the Jalaur River, which is the main source of irrigation water for the neighbouring municipalities. The water hyacinth used in charcoal production was collected from one of the rivers, PD Morfort North River, at 10° 51' 42" North 122° 43' 1"East. According to local regulations, no specific permissions were required to sample in PD Morfort North River. The field sampling did not involve endangered or protected species.

The collected raw materials were brought to the University of the Philippines Visayas, Plant Nursery Station of the Emerging Interdisciplinary Disciplinary Research where the samples were thoroughly washed with distilled water, the plant material excluding the roots were chopped into smaller pieces and sun dried inside the nursery for two weeks. The dried materials were further cut to approximately a length less than 1 cm and width less than 0.5 cm in preparation for the carbonization process.

### Experimental design

**Fig 1:**
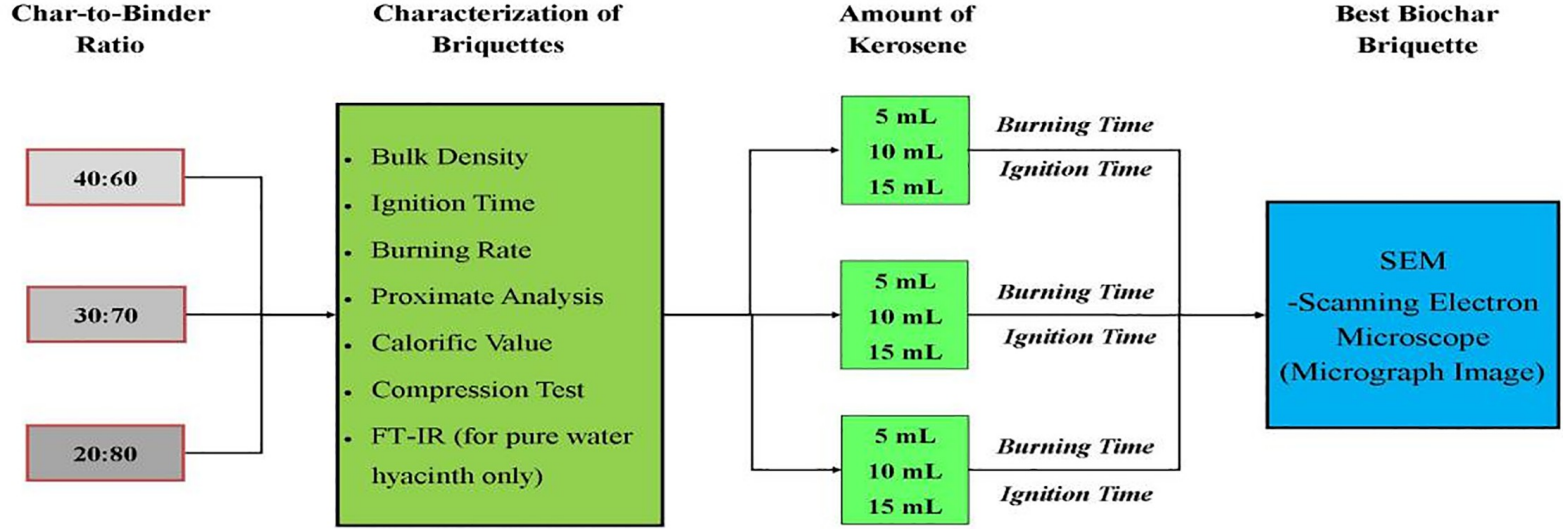
Schematic flow of experiment procedure showing the char/binder ratios used, and characterization of the briquettes produced.

The experiment was divided into four phases: (1) Carbonization of water hyacinth (2) Preparation of the water hyacinth- molasses blends using different charcoal/binder ratios 3) Densification/Briquetting 4) Characterization of the three most well-formed briquettes by proximate analysis, calorific value, maximum compression load, bulk density, burning rate and ignition time. The steps involved in the development of the briquettes by varying char-binder ratio are shown in Fig 1.

### Carbonization of water hyacinth

The dried samples were carbonized at an average temperature of 425°C in a fabricated fine biomass carbonizer designed by the Forest Products Research Development Institute, Department of Science and Technology. The surface temperature of the carbonizer was monitored using a thermocouple (DIGI-SENSE Model 20250–19). The carbonized materials were harvested at an average rate of 36.67g/min (550 grams in 12 minutes). They were immediately removed from the carbonizer and then stored in a closed steel container to cool and prevent the charcoal from ashing.

### Preparation of water hyacinth-molasses blends and briquetting

The binder used was molasses obtained from First Farmer’s Holding Corporation (FFHC), an agro-industrial business enterprise. Various preliminary trial runs using different concentrations of molasses-water solutions were investigated to determine the amount of molasses sufficient to bind with water hyacinth charcoal. The charcoal was then homogeneously mixed manually at different ratios with the molasses solution until a dense mass, with the ability to be compacted, was obtained. From several trials, three charcoal-binder blends with the following water hyacinth-molasses (80% molasses by weight) ratios were chosen for characterization: A (40:60), B(30:70) and C(20:80).

### Briquetting and drying

A pillow-shaped briquette moulder with dimensions (cm) 50 x 60 x 25 was fabricated [21]. Forty grams of the prepared water hyacinth-molasses blends was placed into each mould of the fabricated moulder. In the absence of a hydraulic press, a hydraulic car jack was used for compacting the solids. The pressure applied to each mould (120 psi or 8.27 bar) was set constant by allowing the jack to travel the same distance from the reference to the final point. The manufactured briquettes were dried in a natural convection oven at 105° C for 8 hours, cooled and packed in polyethylene plastics to prevent re-adsorption of water.

### Proximate analysis

Moisture content analysis, volatile combustible matter and ash were determined according to the procedure ASTM D1762-84. For each type of blend A, B and C, one gram of sample in a crucible was dried in a natural convection oven (Binder (ED)-model 115) at 105° C for 2 hours taking note of the initial and final weights using an analytical balance (Shimadzu electronic balance type ATY124). This procedure was repeated until constant weight was obtained. Moisture was then calculated. The sample was further heated at 470°C for 2 hours before the sample turns into ashes in order to calculate the volatile combustible matter. Later, the sample was placed in a furnace (Thermolyne, Thermo Scientific Furnace) and heated at 750°C for 4 hours. The resulting ash was placed in a desiccator, allowed to cool at room temperature and weighed. This procedure was repeated until the weight of ash became constant. Fixed carbon was obtained from the initial weight of the sample minus the humidity, ash and volatile combustible matter.

### Calorific value (HHV)

The calorific value was determined according to the method ASTM n: D5885 –10a method was used. One gram of sample was pelletized, placed in a sample holder (crucible) then transferred to a steel capsule from the diabatic bomb calorimeter (32-11-CI-006, Model no. 6725).

### Bulk density

Using the analytical balance (Shimadzu electronic balance type ATY124), ten samples for each briquette blend were weighed. The volume of the each briquette was solved using the equation [21],

V=w3(hπw−0.142(1−10−hw))

where:

V = the volume of the pillow shaped briquette

h = is the longer side of the briquette

w = is the shorter side of the briquette

### Compression test

Compression test was done in accordance with ASTM D642 (Method for compression test for shipping containers) and TAPPI T811 (Edgewise Compressive Strength for Corrugated Fiberboard). The equipment used was INSTRON Model 1000. Compression Test was conducted at the Packaging Laboratory of Central Philippines University, Iloilo City Philippines.

### Ignition test and burning rate

The burning rate of the bio charcoal briquette is the mass of the biocharcoal briquette burned per unit time. Using match sticks, the samples were ignited one-by-one and the time it takes to burn a certain amount of briquette was recorded. The difference between the initial and final weights of the charcoal is the mass of the charcoal burned. Three varying volumes of kerosene, 5 ml, 10 ml, and 15 ml were added to the briquettes (3 replicates per blend) and the first sign of visible igniting was the basis for recording the ignition time.

### FTIR (Fourier Transform Infrared Spectroscopy)

Infrared spectra of carbonized pure water hyacinth were measured on AVATAR 330 Fourier Transform infrared (FT-IR) Spectrophotometer. The Fourier Transform Infrared Spectroscopy (FT–IR) was done by the Analytical Laboratory Services of Chemistry Department at College Arts and Sciences, University of the Philippines Visayas. Three replicate samples were analysed.

### Scanning electron microscopy

The micro-structure of the water hyacinth charcoal and the briquette considered to possess the best combustion characteristics were analyzed by Scanning Electron Microscopy (SEM) at the Analytical Laboratory of Southeast Asia Fisheries Development Center SEAFDEC). The samples were first transferred to capsules and coated with Palladium (Pd) at 30 mA and analyzed in a JEOL JFC-5510LV Scanning Electron Microscope.

### Statistical analyses

A one-way ANOVA was conducted in order to test the difference for the parameters in briquettes produced with different charcoal/binder ratios. The results were considered to be statistically significant when P<0.05.

## Results and discussion

### Physico-chemical characterization of water hyacinth charcoal

The yield of water hyacinth charcoal at an average carbonization temperature of 425 ^o^C was 55%. According to Antal et al. [22], a practical method for manufacturing high-quality charcoal from biomass realizes near-theoretical yields of 42−62% while traditional methods for charcoal production in developing countries realize yields of 20% or less, and modern industrial technology offers yields of only 25−37%. The yield obtained from this study is in agreement with the expected theoretical yield but higher than the yield from traditional methods of charcoal production. Charcoal yield depends greatly on the range of temperature for the production of pyrolysis products. Demirbas et al. [23] reported that charcoal yield decreased from 43.5 to 31.0% for the walnut shell and 38.3 to 25.4% with an increase in temperature from 550 to 1150 K.

**Fig 2:**
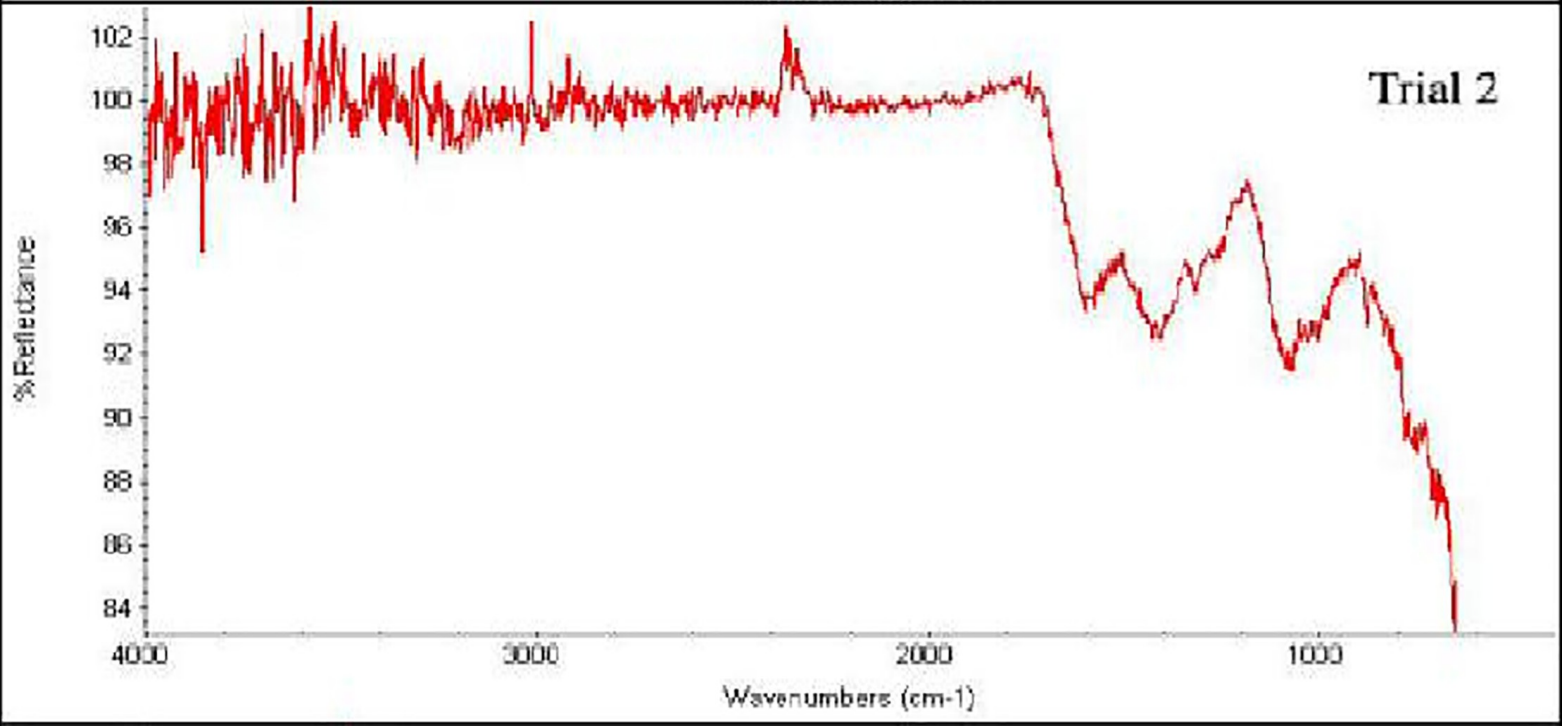
FTIR spectrum of water hyacinth charcoal carbonized at 350–500 ^o^C.

The FTIR peaks (Fig 2) from 760–2500 cm^-1^ of pure water hyacinth charcoal show = C-H, -OH-, C-N, -C = C-, N-H, C-O, C = O, and N-O bonds. The atomic groups and structure present are aromatic, aliphatic, saturated ethers, amines, nitro, tertiary and secondary hydroxyl structures. The presence of aliphatic and aromatic hydrocarbons in the charcoal means that it contains fats and oils that are related to butane or isobutene, making it easier for the charcoal to burn or heat up. Likewise, the presence of hydroxyl groups means that there is an alcohol present which could contribute to higher flammability of substances. The presence of these compounds is an attribute of charcoal having good combustion characteristics.

**Fig 3:**
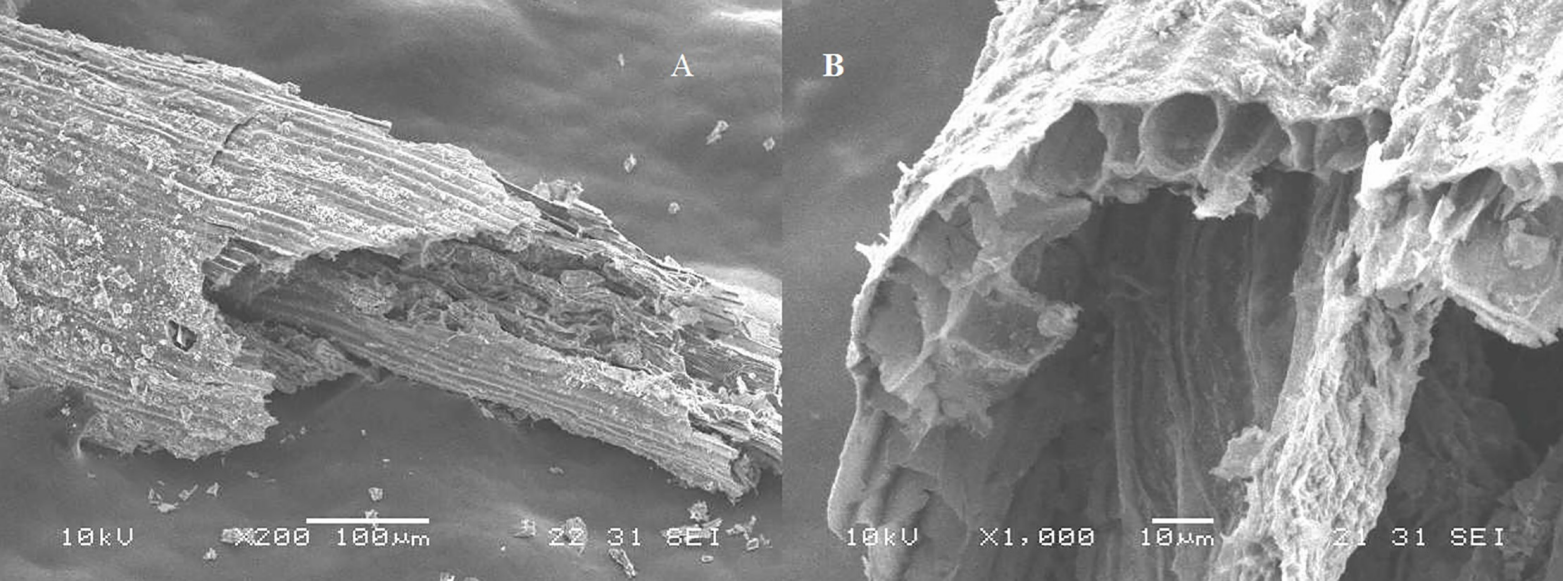
The lengthwise (A) and crosswise (B) SEM images of pure water hyacinth charcoal at 200 and 1,000 magnification.

SEM patterns reveal the porous structure of pure water hyacinth charcoal (Fig 3). The radial image (A) shows rough and stacked features while the cross section reveals an irregular shaped surface exhibiting pores of different sizes. The porous structure both in radial and cross sections can help increase burning efficiency of the charcoal because they provide more paths for airflow allowing more oxygen and air to circulate inside of the charcoal [24].

### Binder characteristics

The molasses and its analyses were provided by First Farmer’s Holding Corporation. The molasses had a brix of 82.38° and total sugar as invert (TSAI) of 56.71%. Total sugar as invert refers to the disaccharides that are inverted by hydrolysis to form monosaccharides. The analysis shows that molasses contain large amount of carbon-containing compounds in sugar thereby affecting the proximate analysis of the briquettes and its combustion property. The molasses was highly viscous, a characteristic required from a good binder in the briquetting technology.

### Characterization of the briquettes

**Fig 4:**
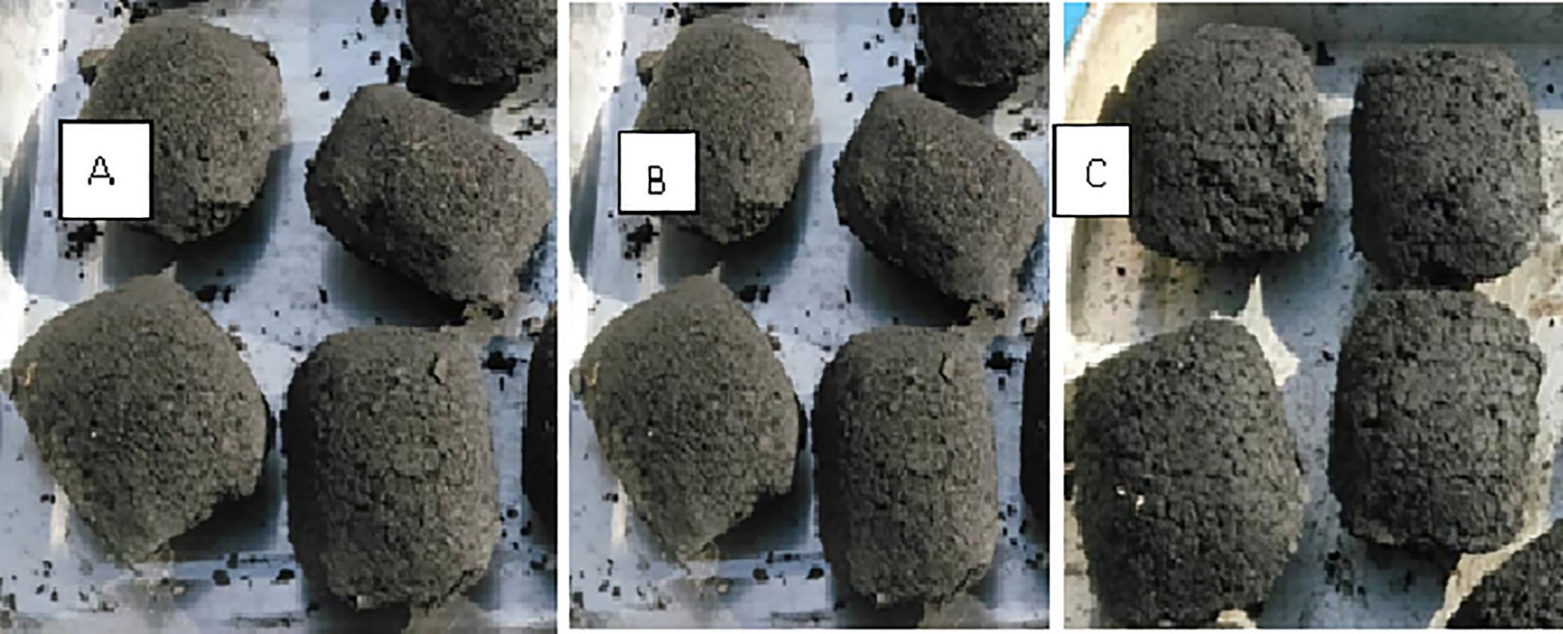
Briquettes produced from different water hyacinth charcoal and molasses ratios. Charcoal/molasses ratio: A (40:60), B(30:70), C (20:80).

The briquettes produced from the different formulations of water hyacinth charcoal and molasses are shown in Fig 4. Briquette A containing 60% by weight molasses solution had smooth edges with no visible cracks indicating that the amount of molasses added was enough to evenly coat the surface. Increasing the amount of molasses to 70% (B) allowed the binder to move deeper into the porous surface and occupy pore spaces within the biomass charcoal. The surface was rougher than that of A. Cracks were observed on the surface and some broke during briquetting. The briquettes with ratio 20:80 (C) appeared weak and crumbly.

**Fig 5:**
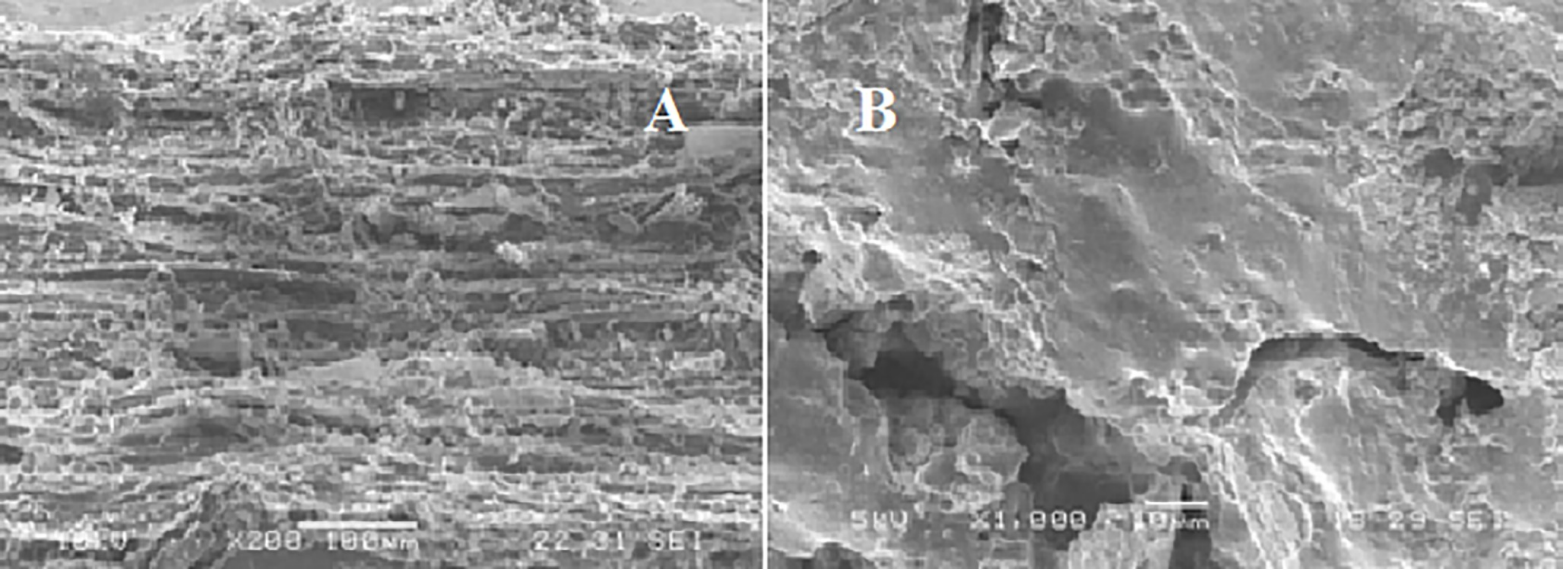
SEM images of water hyacinth charcoal (A) and water hyacinth briquette (B).

Surface images (Fig 5) show distinct morphologies between the charcoal and the briquette. The water hyacinth charcoal (A) had porous rough surface arranged uniformly as stacked layers. Image B shows the level of coating by molasses. The surface were smoother, covered pores are no longer distinguishable but the presence of holes beneath the coatings could still act as air passage and aid in the combustion process.

Table 1 shows the proximate analysis of the formulated water hyacinth-molasses briquette in comparison with briquettes from other biomass and binder sources. The moisture content increased with increasing binder-charcoal ratio. It should be noted that the binder is 80% solution of molasses in water. Greater amount of water was added to briquette C than in B and A, resulting in higher amount of moisture. Moisture content in charcoal is considered as an impurity and could lower the heating value of the charcoal [25]. This is also evident in the briquettes produced. The lowest heating value was found in the briquette with the highest % moisture. Increase in moisture content decreased the heating value since not all the energy contained in the charcoal can be efficiently transferred [26]. According to previous studies, charcoal should have moisture content of 5–15% of its gross weight and charcoal with high moisture content (>10%) becomes brittle when heated [25]. Table 1 shows that briquettes with low moisture content specifically those with woody biomass have higher heating values.

The liquids present in the charcoal other than water which are easy to vaporize are called volatile matters (VCM). Table 1 shows the effect of water hyacinth (WH)-molasses ratio on the VCM content of the briquettes. As the ratio of WH to molasses decreases, the volatile matter content decreases. This result agrees with the findings of Rezania et.al. [15] in their production of briquettes using water hyacinth, empty fruit bunches (palm oil mill residue) and cassava starch. It is important to note from Table 1 that the VCM of pure WH is higher than the VCM of the briquettes produced.

High fixed carbon content of a charcoal means it is made up mostly of carbon. In this study, as the ratio of molasses to WH increases, the fixed carbon content of the briquette also increased. As previously noted, molasses used has a brix of 82.38° and total sugar as invert (TSAI) of 56.71%. This could have contributed to more carbon atoms in briquette C (20:80 ratio of WH to molasses). Compared with other briquettes produced from other sources, FC in this study was comparatively lower than those produced from woody sources and agricultural residues (see Table 1).

The proximate analysis showed that the briquettes having higher fixed carbon have lower ash content. Also, the ash content was found to decrease as the ratio of molasses to WH increased. The amount of ash or the residue is correlated with the amount of fixed carbon and other combustible component of briquette. The fewer residues left after combustion, the greater amount of fixed carbon and combustible substance such as the VCM are present. Ash from woody materials are much lower than the briquettes from water hyacinth (Table 1).

Calorific value is the measure of energy released by the fuel during combustion, while the amount of fixed carbon is one of the major contributors to the heating value of charcoal. Based on the results of this study, briquettes B and C would likely have the greater heating value because of their high FC compared to A. This study however showed that briquette B has the highest heating value. This implies that other factors like the quality of the charcoal, and components like moisture and ash could contribute to the decrease in calorific value. Several studies indicate that high ash content in briquettes will lower calorific value and combustion efficiency [7, 27]. This is in agreement with the results of this study which showed that the high ash content of the briquettes (greater than 15%) resulted in low heating value (< 20 MJ/kg)

The effect of charcoal/binder ratio on bulk density, compressive strength, burning rate and ignition time is shown in Table 2. Bulk density affects combustion efficiency and durability of the briquettes. The denser the material, the easier for it to be transported, handled and stored. Consumers prefer more dense charcoal. The initial failure criterion during compression test was breakage. The average deflection height before breakage of briquette A was 3.47 mm, while that of B was 6.56 mm. The addition of molasses as binder increased the resistance of the briquette to breakage. However increasing further the amount of binder as in C, the sample became malleable and no deflection was obtained. Instead of breaking, C flattened as load was increased to the maximum machine capacity.

**Table 2:** Effect of charcoal/binder ratio on bulk density, compressive strength, burning rate and ignition time.

Characteristic	Treatment			P (0.05)*
	A (60:40)	B (70:30)	C (80:20)	
HHV (MJ/kg)	15.9 ± 3.9 a	16.6 ± 2.1 a	13.4 ± 1.6 a	0.339
Bulk Density (g/cm^3^)	0.89 ± 0.06 a	0.84 ± 0.02 b	0.85 ± 0.02 b	0.042
Compressive Strength (kg/cm^2^)	3.9 ± 0.2 a	19.1 ± 0.5 b	7.4 ± 0.4 c	0.000
Burning Rate (g/s)	0.010 ± 0.001 a	0.009 ± 0.001 a	0.007 ± 0.002 a	0.062
Ignition Time (s)	175 ± 9 a	133 ± 7 b	198±8 c	0.000

*One Way Anova at 95% Confidence (p = 0.05)

The next index of failure considered was the first appearance and formation of cracks upon subjection to load. A was the first to exhibit cracking from a load of 3.93 kg, followed by C which started cracking upon subjection to 7.4 kg. Briquette B showed the maximum compressive strength since cracking occurred at a much higher load of 19 kg. According to M’Ndegwa [28], molasses improve the adherence of particles and forms strong inter-particle bonds between particles thereby enhancing stability of the material.

The results of compressive strength test shows that the 30:70 (B) charcoal to binder ratio has the highest compressive strength followed by 20:80 (C) and the lowest was that 40:60(A). Due to the molasses’ hygroscopic properties, moisture can be trapped inside the briquette. High malleability of C was the effect of greater amount of molasses mixed with the charcoal. Increasing the amount of molasses added to the charcoal caused greater gluing effect but increased the amount of moisture. The increase in binder/charcoal ratio (B) could make the briquette less resistant to cracking but further increase as indicated in C had made the briquette malleable. During transport and handling, briquette C is more resistant to breaking than B but less resistant to cracking. Treatment A showed weak resistance both to breakage and cracking. From Table 2, bulk density and compressive strength have shown inverse relationship with each other. Results show that the briquette with the lowest bulk density which is B (30:70) has the highest compressive strength. While A(40:60) whose bulk density was highest, had the lowest compressive strength.

It is important to understand the factors that affect burning rate and ignition time of biomass or agro-waste briquettes for their more efficient utilization as fuel.

It is shown in Table 2 that burning rate of briquettes decreased with increased binder proportion (C < B < A). Davies and Abolude [29] reported the same findings in the burning of sawdust briquettes and with palm oil sludge as binder. Density has been reported as a parameter that can influence the combustion rate and is characterized by low porosity and reduce the infiltration of oxidant and outflow of the combustion products during combustion [30]. Since incombustible ash has low thermal conductivity, it might also slow down the flame propagation in clay binder. According to Oladeji [31], the density influences the flame propagation in briquettes; fewer free spaces for mass diffusion (low porosity) hinder drying, devolatization and burning. Reduction in porosity and consequently increasing density can influence the combustion rate of briquettes by hampering the outflow and infiltration rate of oxidant during combustion.

A combustible material should be easily ignited, particularly for household consumption. Three varying amounts of kerosene were added to the charcoal briquettes to determine the most suitable hold up volume to be used in the ignition test. The addition of 5 ml per briquette was observed insufficient and did not allow the briquette to ignite easily. Addition of 10 ml of kerosene resulted in an immediate and easier ignition with a matchstick. The 15 ml volume of kerosene indicated excess amount of the liquid oil which cannot be contained by the briquettes. The briquettes became very wet and excess kerosene leaked out, thus no ignition test was conducted with this rate of kerosene. Briquettes were consequently tested with 10 mL of kerosene. Briquette B with 30:70 char-binder ratio exhibited the fastest ignition, followed by A and then C. The differences in ignition time are brought about by differences in densities, porosities and amount of moisture in the briquette. C which has greater moisture content than A and B was the slowest to ignite despite higher porosity than the other two. Treatment B easily ignited because its low density and higher porosity provided more pathways for air to flow thereby increasing ignition efficiency.

Further observation during the ignition test was that treatment B continued to ignite and burn on its own, while treatment A and C stopped igniting after some time.

**Fig 6:**
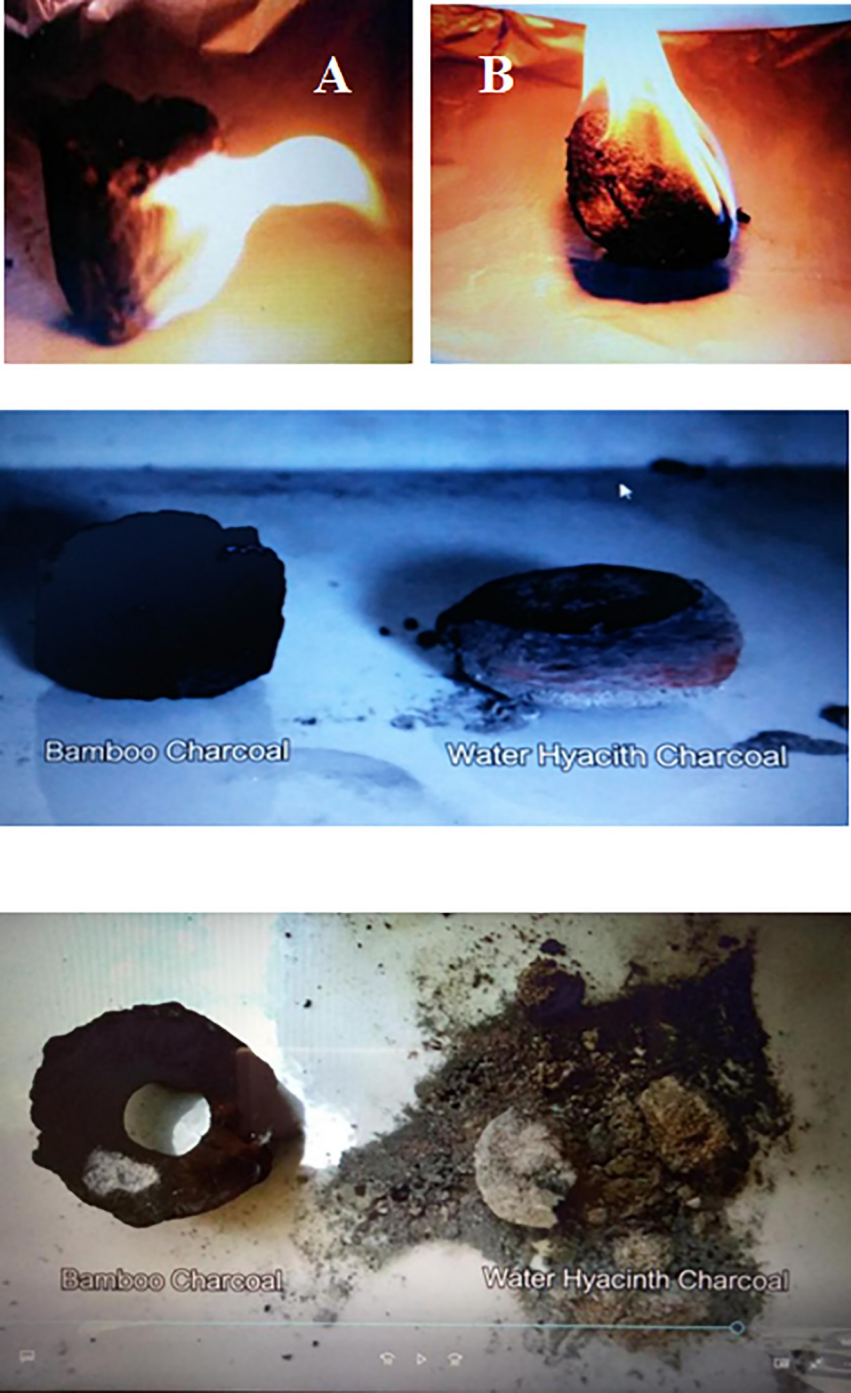
Comparison of the combustion characteristics of water hyacinth briquettes and briquette from bamboo charcoal with cassava as binder.

The burning characteristics of the developed pillow-shaped briquette from water hyacinth and molasses (B) was compared with locally available briquette produced from bamboo and cassava starch (A) from the time of ignition until end of burning (Fig 6). It was observed that bamboo briquette remained on fire for 137 sec and then completely stopped burning. Water hyacinth briquette was on fire for 216 seconds and continued burning until all the solids had burned. Bamboo briquette did not burn completely. The results of our ignition test are similar to those found by other authors [32], who report ranges from 83 to 138 seconds.

Charcoal briquettes should aim to be environmentally acceptable. Studying the overall environmental performance of these materials was outside the scope of this work. Further research could aim to estimate the gas emissions of the briquettes in order to provide an indication of environmental friendliness, similar to studies available on coal-water slurries [33, 34]. For example, previous studies [35] have demonstrated that NOx and SOx emissions can vary dramatically depending on fuel additives. The economic performance [36] of adding binders to the briquette should be considered in conjunction with pondering the environmental advantages.

## Conclusions

In this study, a novel water hyacinth briquette with molasses as binder was developed at varying charcoal/binder. Increasing the amount of molasses as binder increased the moisture content, volatile matter content, and fixed carbon content of the bio charcoal but decreased ash content. The briquette with charcoalto binder ratio of 30:70 showed desirable characteristics in terms of compressive strength, calorific value and ignition. The briquette with 30:70 ratio also showed the highest resistance to breakage with a maximum tolerable load of 19.1 kg/cm^2^, quickest ignition time of 133 seconds and has the highest high heating value of 16.6 MJ/kg. In sum, the developed briquette could be used as fuel in rural areas, and its production could help intervene and alleviate the environmental problems caused by this highly invasive weed in water bodies.
